# Avian Influenza Virus Detection Rates in Poultry and Environment at Live Poultry Markets, Guangdong, China

**DOI:** 10.3201/eid2603.190888

**Published:** 2020-03

**Authors:** Kit Ling Cheng, Jie Wu, Wei Ling Shen, Alvina Y.L. Wong, Qianfang Guo, Jianxiang Yu, Xue Zhuang, Wen Su, Tie Song, Malik Peiris, Hui-Ling Yen, Eric H.Y. Lau

**Affiliations:** University of Hong Kong, Hong Kong, China (K.L. Cheng, A.Y.L. Wong, W. Su, M. Peiris, H.-L. Yen, E.H.Y. Lau);; Guangdong Provincial Center for Disease Control and Prevention, Guangdong, China (J. Wu, W.L. Shen, Q. Guo, J. Yu, X. Zhuang, T. Song)

**Keywords:** avian influenza, live poultry markets, sampling strategy, surveillance, influenza, viruses, China

## Abstract

We report the use of environmental samples to assess avian influenza virus activity in chickens at live poultry markets in China. Results of environmental and chicken samples correlate moderately well. However, collection of multiple environmental samples from holding, processing, and selling areas is recommended to detect viruses expected to have low prevalence.

Live poultry markets (LPMs) can serve as hubs for avian influenza virus (AIV) amplification in poultry and pose a risk for human zoonotic infections (*1*–*4*). Adopting efficient sampling strategies to monitor AIVs with human zoonotic potential at LPMs is essential for zoonotic disease prevention and pandemic preparedness. Recommendations regarding routine surveillance that would robustly and efficiently inform AIV activity at LPMs have been limited (*5*).

Handling of live poultry interrupts the vending process; moreover, such routine surveillance is difficult to implement. Environmental samples have been collected to monitor AIV activity at LPMs (*5*–*9*). There have been limited parallel comparisons of AIV detection rates among poultry and environmental samples (*7*,*10*). Without frequent cleaning, the environment often permits AIV accumulation; environmental samples may thus overestimate AIV prevalence in poultry. Subtype-specific detection rates among different environmental samples may also vary. To inform the development of effective sampling strategies for AIV surveillance, we compared monthly detection rates for AIV subtypes H5, H7, and H9 in chickens and various environmental samples at LPMs in Guangzhou, China.

## The Study

During December 2015–July 2018, we performed sampling twice per month at 1 wholesale (52 stalls) and 1 retail (8 stalls) LPM, from 2 randomly selected stalls per sampling event. We collected paired oropharyngeal and cloacal swab samples (n = 3,119 chickens) and environmental samples (n = 3,008) in viral transport medium at the LPMs (Appendix Figure 1). We randomly collected samples from all chickens at the selected stalls. We rarely observed sick chickens but we sampled them when identified. We also collected environmental samples from 3 key activity areas: poultry holding zones (fecal droppings, drinking water, and poultry feed), slaughtering zones (defeathering machines and surrounding defeathering working areas), and selling zones (chopping boards and display tables) near the selected chickens whenever possible (*5*–*9*). (Stalls sampled at the wholesale LPM [wLPM] have only poultry holding zones.) We sampled air using BC-251 cyclone-based NIOSH bioaerosol samplers that fractionate airborne particles into >4 μm, 1–4 μm, and <1 μm size fractions (*11*). We applied quantitative real-time reverse transcription PCR to detect the matrix gene segment of AIV; we analyzed positive samples by the hemagglutinin gene to determine the AIV subtype (H5, H7, or H9) using specific primers and probes (*12*,*13*).

**Figure 1:**
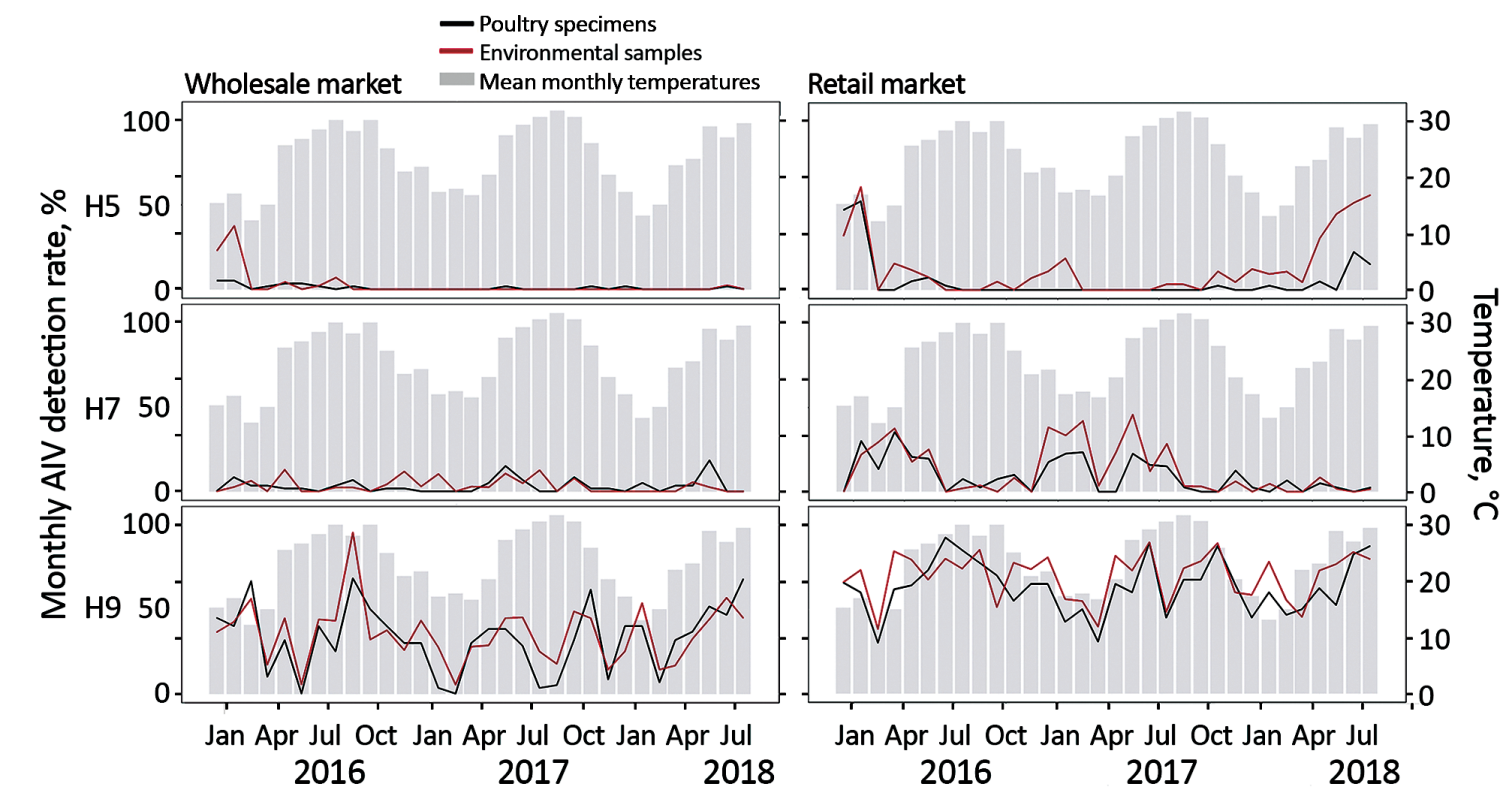
Monthly AIV H5, H7, and H9 positivity rates detected in poultry and environmental samples at live poultry markets (LPMs), Guangdong, China, December 2015–July 2018. Chicken (oropharyngeal and cloacal swab specimens) and environmental (swab specimens and air samples) samples were collected monthly from 1 retail and 1 wholesale LPM in Guangzhou and tested for H5, H7, and H9 AIV by real-time RT-PCR. Gray bars indicate mean temperatures recorded on the sampling date in Guangzhou. AIV, avian influenza virus.

H5, H7, and H9 detection rates in environmental samples (median monthly difference 6.2% for H5, 3.1% for H7, and 34.1% for H9; all p<0.02 by Mann-Whitney test) were much higher at the retail LPM (rLPM) than that at the wLPM (Figure 1), probably because of poultry mixing, aggregation, and extended stay at retail settings. Human H5 or H7 zoonotic infections clustered in winter, but we observed no correlation (p>0.215 for both) between temperature (*14*) and H5 or H7 detection rates in chickens or environmental samples at both markets. We did not assess other confounding factors, including market interventions and poultry holding duration.

We evaluated correlations between monthly AIV detection rates in chickens and environmental samples (moderate correlation for r_s_>0.5, at which point environmental samples are considered useful to monitor AIV in chickens). We observed a positive correlation for H5 (Spearman r_s_ = 0.569, p<0.001) and H9 viruses (r_s_ = 0.702, p<0.001) at the wLPM and for H5 (r_s_ = 0.581, p<0.001), H7 (r_s_ = 0.760, p<0.001), and H9 viruses (r_s_ = 0.685, p<0.001) at the rLPM. We examined the use of environmental samples to assess AIV activity in poultry (Table 1). Environmental samples collected at the rLPM provided higher sensitivity in detecting H5, H7, or H9 viruses in poultry than those from the wLPM. Environmental samples were less likely to detect H5 and H7 viruses in poultry at the wLPM than at the rLPM (Appendix Tables 1, 2), possibly because of the low prevalence of infection in birds, a higher poultry turnover rate, and comparatively thorough daily cleaning practices at the wLPM. The lower specificity for H5 at the rLPM may be the result of carryover contamination at the poultry slaughtering area caused by processing birds of other species. The probabilities of accurately detecting the presence or absence of H5, H7, and H9 subtypes in poultry from environmental samples were comparable for the wLPM (68.8%–93.8%) and the rLPM (59.4%–100%) (Table 1). This finding suggests that environmental samples provided a useful indication of AIV activity in chickens at LPMs. Nevertheless, for H5 and H7 viruses at the wLPM, in only 1 month did all environment samples test positive when bird samples were also positive, demonstrating the need to take a wide range of environment samples. 

**Table 1:** Sensitivity and specificity of applying environmental samples to assess AIV activity in poultry, based on monthly AIV detection, Guangdong, China, December 2015–July 2018*

Market type	Subtype	Sensitivity, % (95% CI)†	Specificity, % (95% CI) †	Positive predictive value, % (95% CI)†	Negative predictive value, % (95% CI)†	Accuracy, % (95% CI)†
Wholesale	H5	45.5 (16.8–76.6)	95.2 (76.2–99.9)	83.3 (39.9–97.4)	76.9 (65.8–85.2)	78.1 (60.0–90.7)
	H7	68.4 (43.5–87.4)	69.2 (38.6–90.9)	76.5 (57.6–88.6)	60.0 (41.4–76.1)	68.8 (50.0–83.9)
	H9‡	100 (88.4–100)	0 (0–84.2)	93.8	NA	93.8 (79.2–99.2)
Retail	H5	90.0 (55.5–99.8)	45.5 (24.4–67.8)	42.9 (32.7–53.7)	90.9 (59.6–98.6)	59.4 (40.6–76.3)
	H7	87.0 (66.4–97.2)	66.7 (29.9–92.5)	87.0 (72.3–94.5)	66.7 (38.7–86.4)	81.3 (63.6–92.8)
	H9§	100 (89.1–100)	NA	100 (89.1–100)	NA	100 (89.1–100)

*Test results from reverse transcription PCR on bird samples were assumed to be the standard in the analysis. The results may not be applicable to other surveillance systems with more intensive sampling or accurate laboratory testing. AIV, avian influenza virus; NA, not applicable. †Sensitivity: probability that the environmental samples will test positive when the subtype of AIV is present in chickens on site (true positive rate). Specificity: probability that the environmental samples will test negative when the subtype of AIV is not present (true negative rate). Positive predictive value: probability that the subtype of AIV is present in poultry when environmental samples are tested positive. Negative predictive value: probability that the subtype of AIV is not present in poultry when the environmental samples are tested negative. Accuracy: probability that the presence or absence of AIV in poultry will be correctly determined based on the test results of environmental samples. ‡H9 was detected during every month during the study period in the environmental samples (monthly data can be found in Appendix Table 1). §H9 was detected during every month during the study period in both the poultry and the environmental samples (monthly data can be found in Appendix Table 2).

**Figure 2:**
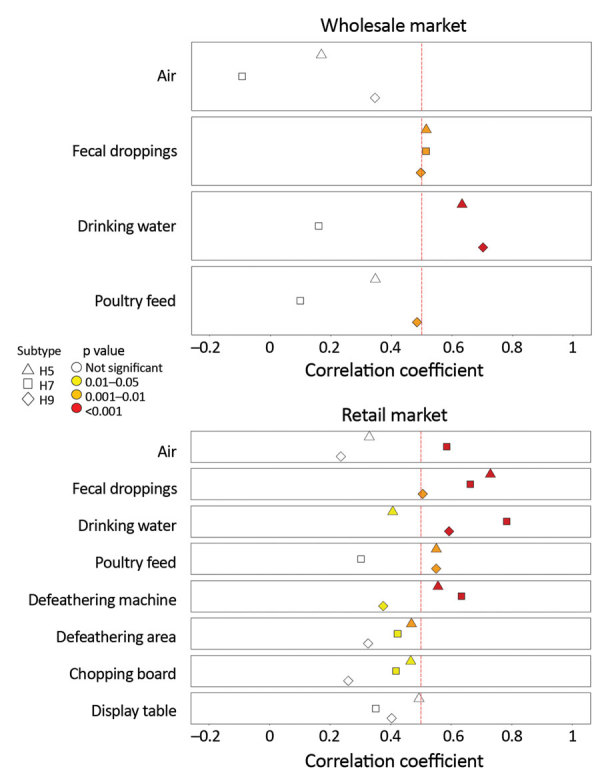
Correlation between AIV detection rates in poultry and environmental samples at live poultry markets (LPMs), Guangdong, China, December 2015–July 2018. Monthly AIV, H5, H7, and H9 detection rates in chicken and environmental samples were analyzed using Spearman’s rank correlation. The vertical red dashed line indicates correlation coefficient (r_s_) at 0.5. Subtypes and significance levels are indicated. AIV, avian influenza virus.

We investigated correlations between specific environmental samples and monthly H5, H7, and H9 detection rates in chickens (Figure 2; Appendix Figure 2). At the wLPM, positive rates for H5 (r_s_ = 0.515, p = 0.003), H7 (r_s_ = 0.514, p = 0.003), and H9 (r_s_ = 0.508, p = 0.003) in fecal droppings correlated moderately well with viral prevalence in chickens, whereas drinking water provided the best correlation for H5 (r_s_ = 0.633, p<0.001) and H9 (r_s_ = 0.702, p<0.001) (Figure 2) and was more sensitive for H9 (Appendix Figure 3). At the rLPM, H9 detection rates in drinking water (r_s_ = 0.593, p<0.001), poultry feed (r_s_ = 0.550, p = 0.002), and fecal droppings (r_s_ = 0.506, p = 0.003) best correlated with H9 prevalence in chickens; drinking water was most sensitive (Appendix Figure 3). H7 detection rates in drinking water (r_s_ = 0.784, p<0.001), fecal droppings (r_s_ = 0.663, p<0.001), defeathering machines (r_s_ = 0.634, p<0.001), and air (r_s_ = 0.585, p<0.001) best correlated with H7 prevalence in chickens. The H5 detection rates in fecal droppings (r_s_ = 0.729, p<0.001), defeathering machines (r_s_ = 0.556, p<0.001), and poultry feed (r_s_ = 0.550, p = 0.02) best correlated with H5 prevalence in chickens. Collectively, these results suggest that fecal droppings may provide a good estimation for H5, H7, and H9 prevalence in chickens at LPMs and that drinking water can be more sensitive in some settings and useful for determining virus contamination in LPMs. For viruses present at low prevalence (e.g., H5), low sensitivity is expected.

We summarized H5, H7, and H9 detection rates in various environmental samples at the rLPM (Table 2). H5 virus was most frequently detected from poultry selling zones (median monthly positive rate 27.9%, 95% CI 0%–50%), especially from chopping boards (33%, 95% CI 0%–50%), whereas H7 virus was most frequently detected from poultry slaughtering zones (6.1%, 95% CI 0%–22.2%), especially from defeathering machines. H9 virus was frequently detected from all sampling sites. However, we found no clear difference in environmental sites for detecting H5, H7, or H9 (Appendix Tables 1, 2).

**Table 2:** AIV detection rates from chicken and environmental samples collected at a retail LPM, Guangdong, China, December 2015–July 2018*

Type of samples	No. samples	Median monthly positive rate, % (95% CI)		
		H5	H7	H9
Poultry samples†	1,239	0.0 (0.0–2.5)	5.8 (0.0–15.0)	64.6 (55.0–67.5)
Oropharyngeal	1,239	0.0 (0.0–2.5)	5.8 (0.0–13.3)	60.0 (52.5–67.5)
Cloacal	1,239	0.0 (0.0–2.5)	0.0 (0.0–2.5)	20.0 (13.2–26.5)
Environmental samples	1,734	6.2 (0.0–11.8)	4.2 (1.6–22.0)	73.8 (60.0–79.4)
Poultry holding zone‡	965	2.9 (0.0–6.3)	3.0 (0–20.0)	68.2 (56.0–75.9)
Fecal droppings	424	0.0 (0.0–7.7)	0.0 (0.0–11.1)	58.3 (50.0–66.7)
Drinking water	364	0.0 (0.0–8.3)	3.3 (0.0–15.4)	83.3 (66.7–100.0)
Poultry feed	177	0.0 (0.0–11.1)	0.0 (0.0–9.1)	50.0 (33.3–70.0)
Poultry slaughtering zone	457	2.2 (0.0–25.0)	6.1 (0.0–22.2)	78.6 (59.5–87.5)
Defeathering machine	250	0.0 (0.0–20.0)	0.0 (0.0–20.0)	86.2 (70.0–100.0)
Defeathering area	207	0.0 (0.0–25.0)	0.0 (0.0–12.5)	73.2 (50.0–87.5)
Poultry selling zone	194	27.9 (0.0–50.0)	0.0 (0.0–14.3)	91.2 (60.0–100.0)
Chopping board	141	33.0 (0.0–50.0)	0.0 (0.0–25.0)	100.0 (71.4–100.0)
Display table	53	0.0 (0.0–66.7)	0.0 (0.0–14.3)	92.9 (25.0–100.0)
Air§	118	0.0 (0.0–25.0)	0.0 (0.0–16.7)	75.0 (50.0–100.0)

*AIV, avian influenza virus; LPM, live poultry market; qRT-PCR, quantitative reverse transcription PCR. †A positive poultry sample may detect AIV in the oropharyngeal samples, cloacal samples, or both by qRT-PCR.  ‡Environmental swab specimens were collected within the same poultry stall at LPMs but may not be from the same cage where the chickens were sampled. Fecal droppings were collected from the ground or cages, drinking water was collected from the water troughs, and poultry feed was sampled from the surface of the bowls or feeders. §A positive air sample may be positive for AIV by qRT-PCR in any of the 3 size fractions collected by a NIOSH sampler (*11*). Two to 6 NIOSH samplers were applied monthly to sample air at the retail markets.

## Conclusions

AIV detection rates in environmental samples correlated moderately with AIV activity in chickens at LPMs. Environmental sampling at rLPMs provides greater sensitivity in detecting H5, H7, and H9 AIV in poultry than that at the wLPMs and should be included as routine surveillance to monitor AIV activity. At the rLPM, H5 and H7 viruses were most frequently detected from poultry selling and poultry slaughtering areas, whereas the highly prevalent H9 viruses were detected frequently at poultry holding, slaughtering, and selling areas. Environmental samples with the highest detection rate for H5, H7, and H9 viruses may not provide the best indication of virus activity in poultry, however. Some market stalls containing viruses with low prevalence would be misclassified if only environmental or bird samples were collected. To detect viruses expected to be present at low prevalence, environmental samples should be collected from multiple sites in each market stall, including samples from holding, processing, and selling areas.

Appendix Additional information about avian influenza virus detection at live poultry markets, Guangdong, China, 2015–2018.
