# Supplementary material for: Avian Influenza Virus Detection Rates in Poultry and Environment at Live Poultry Markets, Guangdong, China
Source: Emerg Infect Dis. 2020 Mar;26(3):591–5. doi: 10.3201/eid2603.190888 (PMC7045814; doi:10.3201/eid2603.190888)
Supplement: Appendix — Additional information about avian influenza virus detection at live poultry markets, Guangdong, China, 2015–2018. [file 19-0888-Techapp-s1.pdf]

Article DOI: https://doi.org/10.3201/eid2603.190888

Avian Influenza Virus Detection Rates in Poultry and Environment at Live Poultry Markets, Guangdong, China

Appendix

Appendix Table 1. Monthly AIV detection rates from chicken and environmental samples collected at the wholesale market during December 2015–July 2018 in Guangzhou, Guangdong, China

		Monthly positive rate (%)					
Year	Month	Oropharyngeal	Cloacal	Fecal droppings	Drinking water	Poultry feed	Air
H5 virus							
2015	Dec	2	3	24	10	50	29
2016	Jan	5	2	40	43	0	0
	Feb	0	0	0	0	0	0
	Mar	0	2	0	0	0	0
	Apr	0	3	4	8	0	0
	May	2	2	0	0	0	0
	Jun	2	0	4	0	0	0
	Jul	0	0	10	0	0	25
	Aug	2	0	0	0	0	0
	Sep	0	0	0	0	0	0
	Oct	0	0	0	0	0	0
	Nov	0	0	0	0	0	0
	Dec	0	0	0	0	0	0
2017	Jan	0	0	0	0	0	0
	Feb	0	0	0	0	0	0
	Mar	0	0	0	0	0	0
	Apr	0	0	0	0	0	0
	May	2	0	0	0	0	0
	Jun	0	0	0	0	0	0
	Jul	0	0	0	0	0	0
	Aug	0	0	0	0	0	0
	Sep	0	0	0	0	0	0
	Oct	2	0	0	0	0	0
	Nov	0	0	0	0	0	0
	Dec	2	2	0	0	0	0
2018	Jan	0	0	0	0	0	0
	Feb	0	0	0	0	0	0
	Mar	0	0	0	0	0	0
	Apr	0	0	0	0	0	0
	May	0	0	0	0	0	0
	Jun	2	0	0	7	0	0
	Jul	0	0	0	0	0	0
H7 virus							
2015	Dec	0	0	0	0	0	0
2016	Jan	5	3	3	0	0	0
	Feb	3	0	0	0	0	29
	Mar	2	2	0	0	0	0
	Apr	2	0	7	25	100	0
	May	2	0	0	0	0	0
	Jun	0	0	0	0	0	0
	Jul	3	0	0	5	0	0
	Aug	7	0	0	6	0	0
	Sep	0	0	0	0	0	0
	Oct	2	0	0	7	0	25
	Nov	2	0	8	11	17	25
	Dec	0	0	0	0	0	25
2017	Jan	0	0	7	9	0	50

Year	Month	Monthly positive rate (%)					
		Oropharyngeal	Cloacal	Fecal droppings	Drinking water	Poultry feed	Air
	Feb	0	0	0	0	0	0
	Mar	0	0	0	0	0	25
	Apr	3	2	5	0	0	0
	May	15	3	7	10	0	50
	Jun	3	3	7	8	0	0
	Jul	0	0	0	8	10	75
	Aug	0	0	0	0	0	0
	Sep	5	3	8	0	17	0
	Oct	2	0	0	0	0	0
	Nov	2	0	0	0	0	0
	Dec	0	0	0	0	0	0
2018	Jan	2	3	0	0	0	0
	Feb	0	0	0	0	0	0
	Mar	0	3	0	0	0	0
	Apr	3	0	0	0	14	25
	May	13	8	8	0	0	0
	Jun	0	0	0	0	0	0
	Jul	0	0	0	0	0	0
H9 virus							
2015	Dec	45	22	20	50	100	57
2016	Jan	38	17	37	57	0	100
	Feb	67	28	43	100	100	71
	Mar	7	3	0	50	33	0
	Apr	30	17	33	83	100	14
	May	0	0	0	0	0	33
	Jun	40	7	44	77	50	0
	Jul	25	0	10	68	0	100
	Aug	68	20	100	100	89	75
	Sep	50	3	5	67	46	0
	Oct	40	13	6	50	50	75
	Nov	30	2	8	44	17	50
	Dec	30	15	31	55	33	75
2017	Jan	3	0	7	46	30	50
	Feb	0	0	0	0	0	50
	Mar	30	3	13	44	29	50
	Apr	38	10	21	39	17	50
	May	38	2	7	80	40	100
	Jun	28	7	36	39	55	75
	Jul	2	2	23	31	10	50
	Aug	5	0	10	20	20	25
	Sep	32	2	17	82	42	75
	Oct	60	15	36	64	0	100
	Nov	7	2	0	21	10	50
	Dec	40	7	8	39	20	50
2018	Jan	40	2	15	92	46	75
	Feb	5	2	0	18	9	75
	Mar	32	2	7	29	0	50
	Apr	37	3	0	39	71	50
	May	52	25	31	54	36	75
	Jun	47	10	7	93	75	50
	Jul	68	2	15	77	22	100

Appendix Table 2. Monthly AIV detection rates from chicken and environmental samples collected at retail markets during December 2015–July 2018 in Guangzhou, Guangdong, China

		Monthly positive rate (%)									
Year	Month	Oropharyngeal	Cloacal	Fecal droppings	Drinking water	Poultry feed	Defeathering machine	Defeathering area	Chopping board	Display table	Air
					H5 virus						
2015	Dec	32	42	32	0	100	33	0	100	50	0
2016	Jan	50	33	54	83	100	100	50	*	*	50
	Feb	0	0	0	0	0	0	0	*	*	0
	Mar	0	0	8	0	*	40	50	*	*	0
	Apr	3	3	0	31	*	0	*	*	*	0
	May	5	5	8	0	0	50	*	*	*	0
	Jun	3	0	0	0	0	0	0	0	*	0
	Jul	0	0	0	0	0	0	0	0	*	0
	Aug	0	0	0	0	0	0	0	*	*	0
	Sep	0	0	0	0	0	0	22	*	*	0
	Oct	0	0	0	0	0	0	0	*	*	0
	Nov	0	0	8	9	0	11	0	100	*	25
	Dec	0	0	0	0	33	13	7	50	*	25
2017	Jan	0	0	0	10	0	20	14	43	100	50
	Feb	0	0	0	0	0	0	0	0	0	0
	Mar	0	0	0	0	0	0	0	0	0	0
	Apr	0	0	0	0	0	0	0	0	0	0
	May	0	0	0	0	0	0	0	0	0	0
	Jun	0	0	0	0	0	0	0	0	*	0
	Jul	0	0	0	15	0	0	0	0	*	0
	Aug	0	0	0	0	0	0	0	29	*	0
	Sep	0	0	0	0	0	0	0	0	0	0
	Oct	3	0	9	0	0	25	25	20	*	0
	Nov	0	0	0	0	0	0	0	38	0	0
	Dec	0	0	0	0	0	10	50	38	0	0
2018	Jan	3	3	8	0	0	0	25	43	0	0
	Feb	0	0	0	0	11	13	25	43	0	0
	Mar	0	0	0	17	0	0	0	0	0	0
	Apr	5	0	25	8	22	30	100	67	0	25
	May	0	0	8	8	44	63	100	100	67	50
	Jun	23	3	8	33	42	83	100	88	100	50
	Jul	15	3	17	43	30	83	80	100	100	75
					H7 virus						
2015	Dec	0	0	0	0	0	0	0	0	0	0
2016	Jan	18	20	25	17	0	50	0	*	*	0
	Feb	13	7	9	44	0	33	43	*	*	50
	Mar	32	15	46	50	*	40	0	*	*	0
	Apr	15	10	24	15	*	0	*	*	*	0
	May	17	12	15	33	50	25	*	*	*	17
	Jun	0	0	0	0	0	0	0	0	*	0
	Jul	8	0	0	7	0	0	0	0	*	0
	Aug	3	0	0	13	0	0	0	*	*	0
	Sep	8	0	0	0	0	0	0	*	*	0
	Oct	10	0	11	0	0	17	0	*	*	25
	Nov	0	0	0	0	0	0	0	0	*	0
	Dec	18	0	11	40	33	53	36	50	*	25
2017	Jan	23	10	23	50	0	20	36	29	50	75
	Feb	17	20	0	33	33	0	20	100	100	100
	Mar	0	0	0	0	0	0	0	25	0	0
	Apr	0	0	17	0	33	39	33	29	50	0
	May	20	3	40	46	0	70	56	50	0	50
	Jun	11	5	0	8	10	14	40	0	*	50
	Jul	15	8	17	46	14	30	50	0	*	25
	Aug	3	0	0	8	0	0	13	0	*	0
	Sep	0	0	0	9	9	0	0	0	0	0
	Oct	0	0	0	0	0	0	0	0	*	0
	Nov	10	3	0	8	0	20	0	13	0	0
	Dec	3	0	0	0	0	0	0	0	0	0
2018	Jan	0	0	0	0	0	0	13	29	0	0
	Feb	7	0	0	0	0	0	0	0	0	0
	Mar	0	0	0	0	0	0	0	0	0	0
	Apr	5	0	8	0	0	10	0	17	0	50
	May	0	3	0	0	0	0	14	0	0	0
	Jun	0	0	0	0	0	0	0	0	0	0

Year	Month	Monthly positive rate (%)									
		Oropharyngeal	Cloacal	Fecal droppings	Drinking water	Poultry feed	Defeathering machine	Defeathering area	Chopping board	Display table	Air
	Jul	3	0	0	0	0	0	0	0	14	0
H9 virus											
2015	Dec	55	45	65	33	100	67	0	100	100	100
2016	Jan	58	43	68	100	100	50	50	*	*	100
	Feb	20	13	27	33	100	67	14	*	*	100
	Mar	59	27	77	100	*	80	75	*	*	100
	Apr	46	41	77	85	*	100	*	*	*	50
	May	73	22	62	93	50	50	*	*	*	33
	Jun	93	35	50	100	100	100	100	100	*	100
	Jul	83	23	53	100	80	60	100	33	*	100
	Aug	73	23	80	100	100	100	50	*	*	50
	Sep	70	18	50	63	50	100	22	*	*	25
	Oct	53	15	33	92	60	92	86	*	*	100
	Nov	65	23	92	73	50	78	64	0	*	100
	Dec	65	10	67	100	33	100	71	100	*	50
2017	Jan	43	8	39	90	0	87	41	29	100	50
	Feb	50	3	0	67	33	50	20	100	100	100
	Mar	31	8	27	36	0	58	60	38	0	75
	Apr	63	30	67	70	83	92	100	100	100	50
	May	60	3	60	73	33	100	56	100	50	75
	Jun	90	13	92	100	70	86	80	100	*	100
	Jul	43	13	17	62	14	60	50	100	*	50
	Aug	68	8	50	83	40	89	88	100	*	100
	Sep	63	15	55	100	91	100	25	88	100	75
	Oct	88	15	82	92	67	100	100	90	*	100
	Nov	63	20	33	75	40	70	100	75	0	50
	Dec	45	5	33	58	33	70	88	100	0	50
2018	Jan	60	25	75	100	70	100	75	71	33	50
	Feb	40	30	50	46	33	75	100	57	25	50
	Mar	50	0	46	56	29	43	33	57	50	25
	Apr	60	38	58	75	56	100	25	100	100	75
	May	53	20	75	67	33	100	100	100	100	75
	Jun	80	33	58	83	83	100	100	100	100	75
	Jul	88	38	58	100	50	83	80	100	86	100

*No samples were collected.

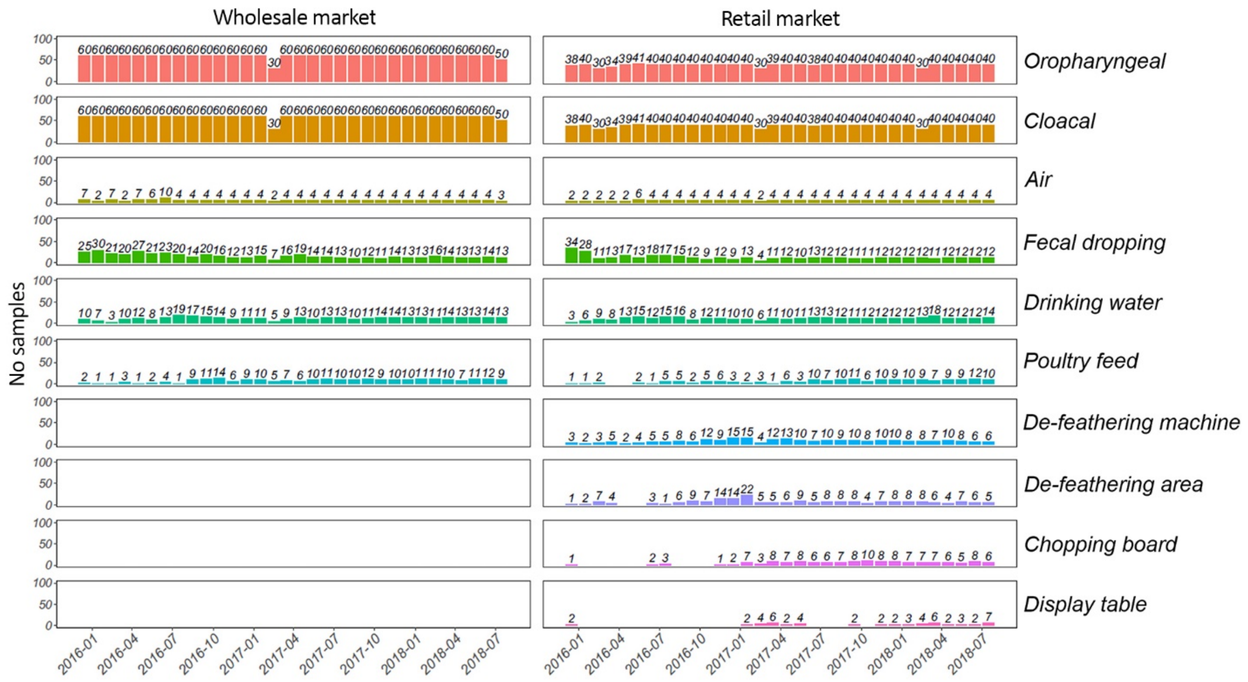

Appendix Figure 1. Number of poultry swabs, environmental swabs, and air samples collected monthly at the wholesale and retail live poultry markets, Guangzhou, China, December 2015–July 2018.

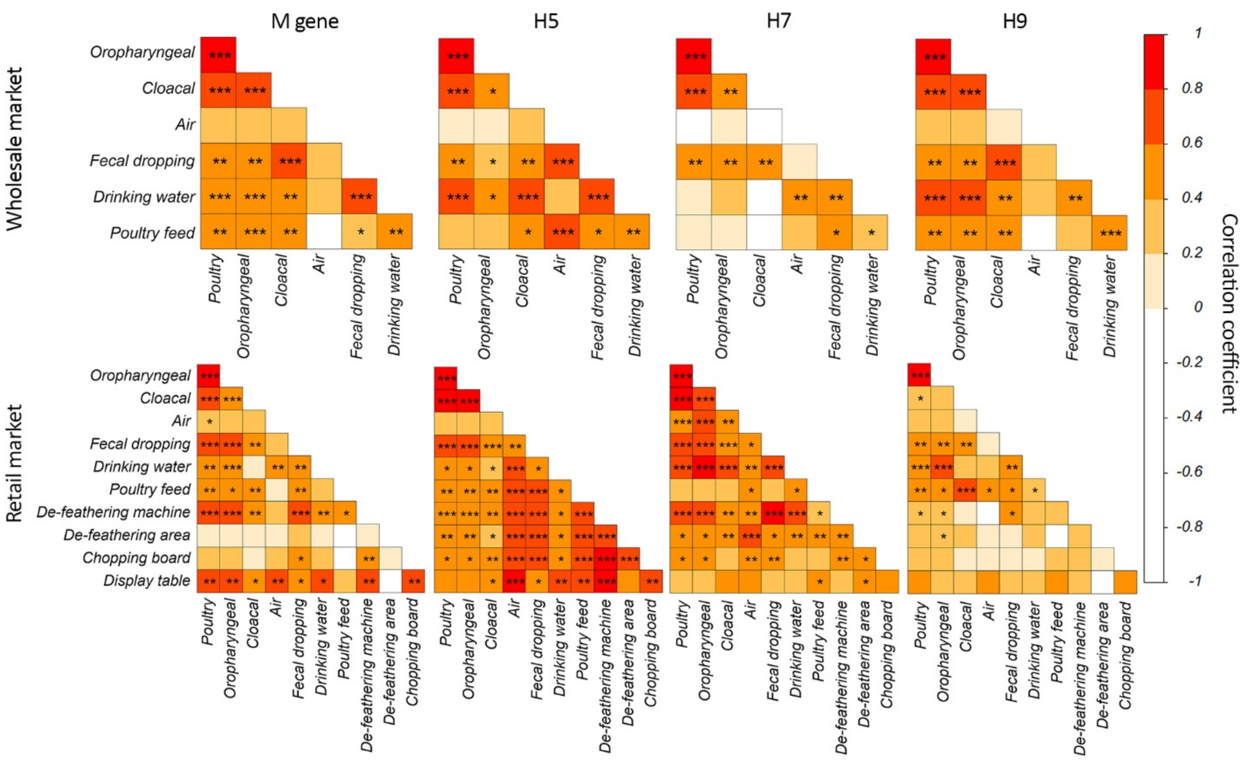

Appendix Figure 2. Correlation matrices for monthly AIV detection frequencies across different sample types. Gradient of shades from light orange to red indicate increasing positive correlation coefficient (r_s) by Spearman’s rank correlation. The significance level is indicated as follows:

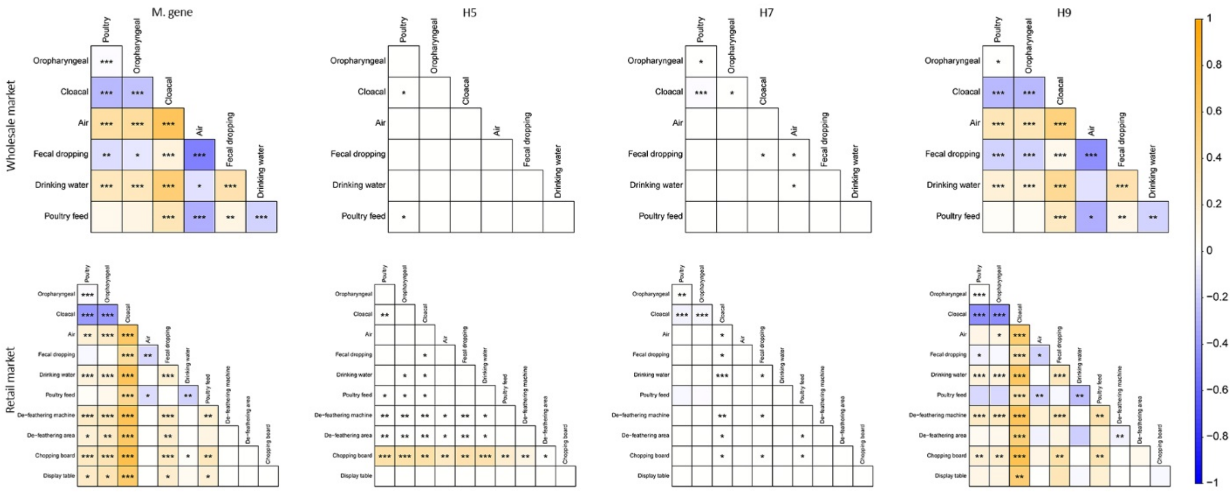

Appendix Figure 3. Differences monthly AIV detection rates across different sample types. Gradient of shades from light orange to red indicate increasing median of monthly difference (detection rates of the sample type shown by the row labels minus that shown by the column labels). The significance level is indicated as follows: *0.01≤p<0.05; **0.001≤p<0.01; ***p<0.001.
